# Supplementary material for: Organic Food in the Diet of Residents of the Visegrad Group (V4) Countries—Reasons for and Barriers to Its Purchasing
Source: Nutrients. 2021 Dec 2;13(12):4351. doi: 10.3390/nu13124351 (PMC8708884; doi:10.3390/nu13124351)
Supplement: Supplementary file 1 [file nutrients-13-04351-s001.zip › nutrients-1418014-supplementary.pdf]

nutrients

MDPI

Attachment S1

Table S1. Questionnaire for organic consumers.

1. Motives to purchasing organic food: 1 – not important, 2 – low important 3 - medium important , 4 – important, 5 – very important.

Specification	Grade				
	1	2	3	4	5
1. It tastes good					
2. Low in calories					
3. Little processed					
4. Lots of nutritional values					
5. High health values					
6. Environmentally friendly					
7. No preservatives					
8. Not genetically modified					
9. Attractive appearance					
10. Does not cause allergies					
11. Free from chemicals					
12. Other, specify…….					

2.Barriers to purchasing organic food: : 1 – not important, 2 – low important 3 - medium important , 4 – important, 5 – very important..

Specification	Grade				
	1	2	3	4	5
1. Too expensive					
2. Not easily available					
3. Lack of trust					
4. Short shelf life					
5. Other, specify…….					

3.How often do you buy organic food

- very rarely
- rarely
- moderately
- often
- very often

4. Your country

- Poland
- Hungary
- Czechia
- Slovakia

5. Your sex

- female
- male

6. Your Age

- from 20 to 34 years
- from 35 to 49 years
- from 50 to 65 years
- 65 years or over

Source: Own study
